# Recapitulating the Drifting and Fusion of Two-Generation Spheroids on Concave Agarose Microwells

**DOI:** 10.3390/ijms241511967

**Published:** 2023-07-26

**Authors:** Rong Pan, Xiaoyan Yang, Ke Ning, Yuanyuan Xie, Feng Chen, Ling Yu

**Affiliations:** Key Laboratory of Luminescence Analysis and Molecular Sensing, Ministry of Education, Institute for Clean Energy and Advanced Materials, School of Materials and Energy, Southwest University, Chongqing 400715, China; prswu2021@email.swu.edu.cn (R.P.); yxy0319@email.swu.edu.cn (X.Y.); nk7868@email.swu.edu.cn (K.N.); blsy486900@email.swu.edu.cn (Y.X.); cf199506@email.swu.edu.cn (F.C.)

**Keywords:** cell spheroids, bio-assembling, spheroid fusion, 3D cell culture, agarose array

## Abstract

Cells with various structures and proteins naturally come together to cooperate in vivo. This study used cell spheroids cultured in agarose micro-wells as a 3D model to study the movement of cells or spheroids toward other spheroids. The formation dynamics of tumor spheroids and the interactions of two batches of cells in the agarose micro-wells were studied. The results showed that a concave bottom micro-well (diameter: 2 mm, depth: 2 mm) prepared from 3% agarose could be used to study the interaction of two batches of cells. The initial tumor cell numbers from 5 × 10^3^ cells/well to 6 × 10^4^ cells/well all could form 3D spheroids after 3 days of incubation. Adding the second batch of DU 145 cells to the existing DU 145 spheroid resulted in the formation of satellite cell spheroids around the existing parental tumor spheroid. Complete fusion of two generation cell spheroids was observed when the parental spheroids were formed from 1 × 10^4^ and 2 × 10^4^ cells, and the second batch of cells was 5 × 10^3^ per well. A higher amount of the second batch of cells (1 × 10^4^ cell/well) led to the formation of independent satellite spheroids after 48 h of co-culture, suggesting the behavior of the second batch of cells towards existing parental spheroids depended on various factors, such as the volume of the parental spheroids and the number of the second batch cells. The interactions between the tumor spheroids and Human Umbilical Vein Endothelial Cells (HUVECs) were modeled on concave agarose micro-wells. The HUVECs (3 × 10^3^ cell/well) were observed to gather around the parental tumor spheroids formed from 1 × 10^4^, 2 × 10^4^, and 3 × 10^4^ cells per well rather than aggregate on their own to form HUVEC spheroids. This study highlights the importance of analyzing the biological properties of cells before designing experimental procedures for the sequential fusion of cell spheroids. The study further emphasizes the significant roles that cell density and the volume of the spheroids play in determining the location and movement of cells.

## 1. Introduction

In the field of cellular biology, organizing cells into three-dimensional (3D) structures has become a powerful tool for studying cellular behavior in more realistic and complex settings [1,2,3,4,5]. The creation of 3D cell culture systems has allowed researchers to investigate the development, differentiation, proliferation, migration of cells, and interactions between cells and extracellular matrix components [6,7,8,9,10]. Cell spheroids are multicellular aggregates that mimic many aspects of in vivo physiology and have become a typical model in 3D cell culture [11,12,13]. Spheroids can be generated from different cell types and have been used in various applications, including drug discovery, toxicology testing, tissue engineering, and cancer research.

Multicellular spheroids are increasingly employed as building blocks to achieve complex shapes [14,15,16], organisms [17,18], and other biomimetic complexities in larger engineered tissues [19,20,21,22] by fusing them together. Moreover, the fusion of spheroids was recognized as one of the bottom-up tissue engineering strategies [23]. The fusion of spheroids can lead to larger and more complex structures resembling tissue assemblies [24,25,26]. For example, Cui et al. used multi-way assembly cell spheroids to investigate the propagation of Wnt signaling within hetero-spheroid 3D architectures [27]. Fleming et al. investigated the ability of uniluminal vascular spheroids to fuse and preserve the morphological architecture of the original uniluminal vascular spheroids by fusing uniluminal vascular spheroids [28]. Liu et al. established scaffold-free airway tubes with predefined shapes by assembling individual airway organoids of different sizes using multi-Organoid patterning and fusion [29]. Those works were conducted by generating spheroids on platforms, such as hanging drops [25,30], agarose-filled micro-well plates [31], non-adhesive microplates [26], pipette tips [32] or agarose microarrays [24,33]. Then two or more spheroids were put together to achieve physical contact, and the subsequent merging or fusion process of the spheroids was monitored. Particularly, the factors that influence the fusion process were not explored in detail. 

This work aims to study the formation and movement of spheroids to assemble larger spheroids. First, the impact of agarose concentration and the agarose micro-wells’ geometry on tumor spheroids formation were systematically studied. Then the interaction between cells and existing spheroids was studied by generating the 1st generation of spheroids (or parental spheroids) in the agarose microwell and subsequently adding the 2nd batch of cells. The behavior of the 2nd batch of cells towards the existing parental spheroids was investigated by tuning the cell density of the two batches. Factors, such as the cell types, the volume of the parental tumor spheroids, and the number of the 2nd batch cells on cell spheroids assembling and fusion were studied. 

## 2. Results 

### 2.1. Impact of Agarose Concentration and Geometrical Structure of Agarose Micro-Well on Tumor Spheroid Formation

**Figure 1:**
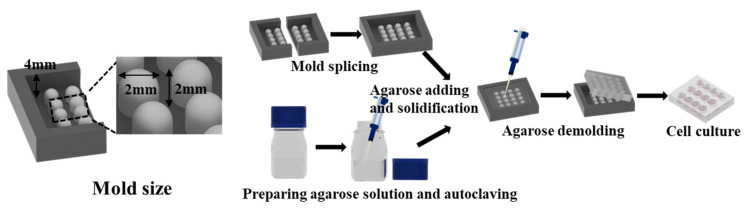
Scheme of the replication method used to create an agarose micro-well array using a 3D-printed mold.

**Figure 2:**
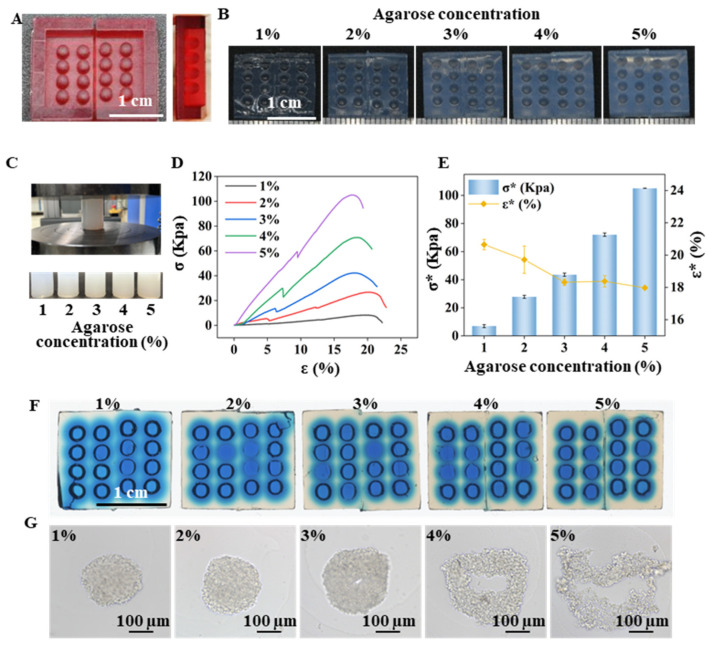
Features of the agarose micro-well for spheroid formation. (**A**) A fabricated 3D-print mold used to generate the agarose micro-well array. Scale bar = 1 cm. (**B**) Micro-well array generated from the different agarose concentrations. Scale bar = 1 cm; (**C**) Compression test and the agarose gel cylinders for the test. (**D**) Stress-strain curve of the agarose gel with different concentrations. (**E**) Breaking strain rate (ε*) and the breaking stress (σ*) of the agarose gel cylinder with different agarose concentrations. (**F**) Blue-dye solution diffusion in the micro-well array generated from the different agarose concentrations. Scale bar = 1 cm. (**G**) Cells cultured on micro-wells generated from the different agarose concentrations for 3 days. Scale bar = 100 μm.

Figure 1 demonstrates the replication method used to create an agarose micro-well array from a 3D-printed mold. The mold contains a 4 × 4 micro-pillar array (Figure 2A) that was divided into two parts to remove the mold from the gelled agarose efficiently. The replicated agarose micro-well array became visually white as the agarose concentration increased from 1% (*w/v*) to 5% (*w/v*) (Figure 2B). The mechanical properties of the agarose gel prepared from different agarose concentrations were characterized by the electromechanical universal testing machine (Figure 2C). The stress-strain curve contains linear and nonlinear parts before the sample was destroyed (Figure 2D). The linear to nonlinear transition denotes the frost breakage, which corresponds to the buckling of the gel. After buckling, a portion of water flowed out of the agarose gel, and the curve transitioned into the nonlinear region. The nonlinear region ended when the gel disintegrated and then the stress dropped sharply. With the increase in agarose concentration from 1% to 5%, the breaking stress (σ*) increased from 6.9621 ± 1.0708 Kpa to 105.0870 ± 0.0668 Kpa. The breaking strain rate (ε*) reached a plateau when the gel was prepared from 3% agarose (Figure 2E). Attempting to separate the 1% and 2% agarose prepared array from the mold proved difficult due to low mechanical strength. When a food color solution was added to the micro-well, it was observed that as the agarose concentration increased, the diffusion speed of the blue solution within the agarose decreased (Figure 2F). Micro-well arrays prepared from different agarose concentrations were used for cell culture, and it was found that cell spheroids were nicely formed in 1%, 2%, and 3% agarose prepared micro-wells but cell clusters with irregular shapes were formed in micro-wells prepared with 4% and 5% agarose (Figure 2G). The slow exchange of the culture medium in the denser gelled agarose base may induce a delay of nutrition supply and, in turn, affect the growth and aggregation of cells. In subsequent experiments, a micro-well array prepared with 3% agarose was used for 3D cell culture.

**Figure 3:**
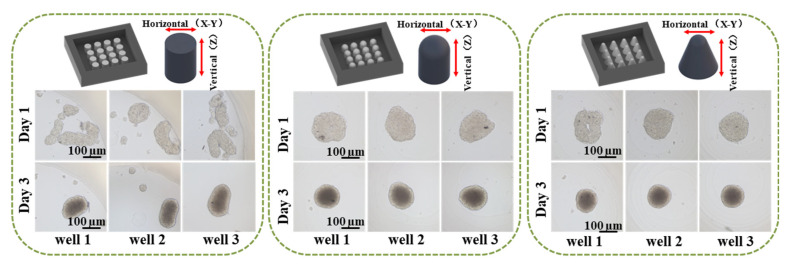
Impact of the geometrical architecture of the agarose micro-well on spheroid formation. Scale bar = 100 µm.

The effect of the micro-well shape on the cell spheroid formation was also evaluated using three types of micro-well geometric structures as seen in Figure 3. The micro-wells have an open width of 2 mm and a height of 2 mm but differ in the half-height width and bottom shape. The micro-wells with a “V” shape bottom have a reduced half-height width, potentially increasing the cell-cell coalescence frequency and decreasing the cell-cell distance. The spheroids with clear edges were formed in sharp bottom micro-wells after a 24 h incubation but not in flat bottom micro-wells. After 3 days of incubation, perfect spheroids were observed at the center of both concave and V-shaped bottom micro-wells, while the cell clusters in flat bottom micro-wells were randomly distributed. The results indicate that the geometrical structure of the micro-wells significantly impacts cell spheroid formation.

**Figure 4:**
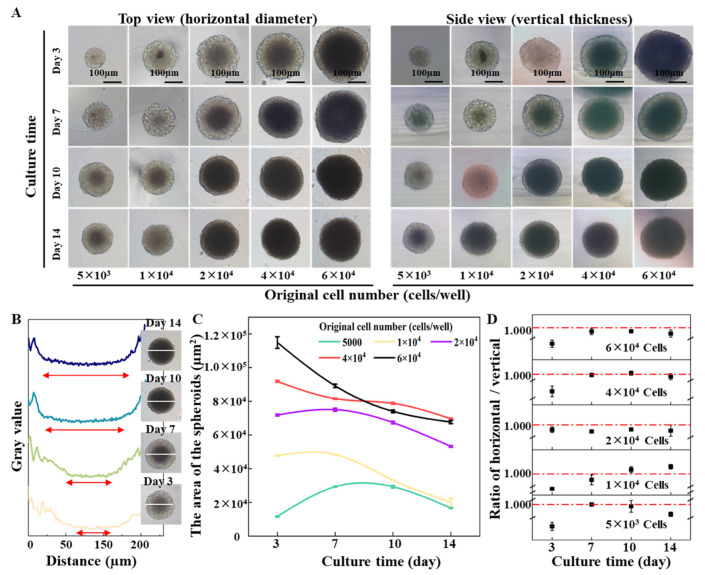
Impact of the initial cell number on spheroid formation and growth in an agarose micro-well. (**A**) Time-lapse top view (horizontal diameter) and side view (vertical thickness) of cell spheroids during 14 days’ growth. Scale bar = 100 µm. (**B**) The profile plot of spheroids formed by 2 × 10^4^ cells during 14 days’ growth. (**C**) The area of spheroids with different initial cell numbers. (**D**) Ratios of horizontal and vertical dimensions of the cell spheroids.

Next, the formation dynamics of the cells in the 3% agarose-prepared concave bottom micro-wells were characterized. The images of the cells in the micro-wells were recorded at different growth periods. The top view shows the spheroid’s horizontal dimension (x-y axis), while the side view characterizes the spheroid’s vertical dimension (z-axis) or thickness. From the top view and side view pictures in Figure 4A, it was found that the initial cell numbers from 5 × 10^3^ cells/well to 6 × 10^4^ cells/well all can form nice spheroids after 3 days of incubation. With the increase in culture time, a dark core can be observed from all spheroids even when starting with only 5 × 10^3^ cells/well. From the profile plot function of ImageJ, the width of the dark core can be quantified (Figure 4B). It was found that spheroids formed from large cell numbers would result in a large dark core. According to the previous studies, the dark core of the spheroids indicates quiescent cells and necrotic core [34,35,36]. Regardless of the starting cell numbers, spheroids do not grow indefinitely. The growth trend of all the formed spheroids can be divided into three phases: rapid growth period, growth slowdown period, and decay period. The difference is that the larger seeding cell number forms a quiescent center early. The smaller the seeding cell number, the longer the rapid growth period is maintained. As shown in Figure 4C, when the original number is 5 × 10^3^ cells/well, the area of the spheroid does not decrease until the tenth day. However, when the number reached 6 × 10^4^ cells/well, the area of the spheroid on the third day already showed a downward trend. In addition, the ratio of the horizontal size and thickness of the spheroids show that “ball” shape spheroids were formed in the micro-well (Figure 4D). The phenome in Figure 4 suggests that when cell density is higher, cell-to-cell contact and interaction will be more accessible because of the space limitation, which facilitates the formation of cell aggregates and spheroids in a short period. From the systemically characterized, it was found that the initial cell number determines the overall size of the spheroids. With the increase in culture time, the thickness of the cell cluster increased, and gradually a ball shape spheroid formed. A longer culture time will result in a larger portion of a quiescent center or a necrosis core, which reflects the pathological condition of a tumor in vivo. 

### 2.2. Cell Spheroids Migrating and Assembling on Micro-Well with Different Geometrical Structures 

**Figure 5:**
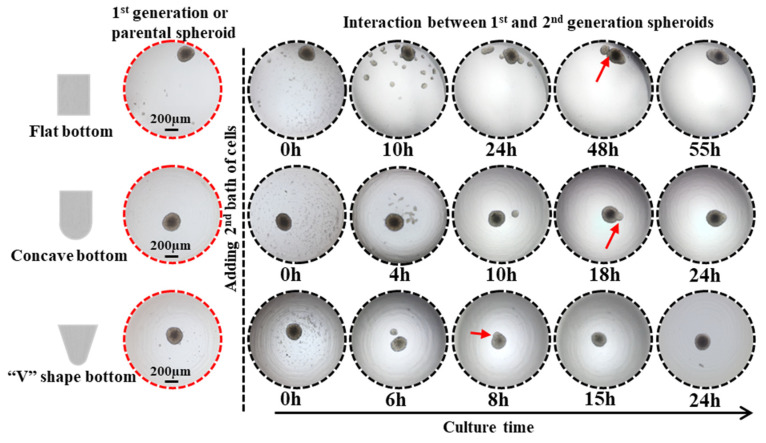
Impact of the geometrical architecture of agarose micro-wells on the interaction of existing parental spheroids and 2nd batch of cells. Red arrow points to spheroid fusion. Scale bar = 200 μm.

The migration and assembly of cell spheroids were investigated using micro-wells with varying geometrical structures. DU 145 cells were utilized as model cells to examine cell movement and attraction. Initially, 1 × 10^4^ DU 145 cells were introduced into the micro-wells, forming cell spheroids after 3 days of incubation. Subsequently, the second batch of cells (3 × 10^3^ DU 145 cells) was added to the micro-wells containing a spheroid. The 2nd batch of cells dispersed uniformly into the culture medium once loaded (Figure 5). Upon analysis of the time-lapse images, we observed that the newly introduced cells tended to aggregate and form numerous tiny spheroids, or satellite spheroids, after 10 h of incubation in flat bottom micro-wells. The tiny cell aggregates merged into a larger cell cluster and eventually migrated and attached to the existing spheroid, forming one large spheroid after 55 h of incubation.

In contrast, in concave micro-wells, some newly introduced cells attached directly to the surface of the existing spheroid after 4 h of incubation. The remaining cells clustered and formed satellite spheroids encircling the parental spheroid, resembling a planet and its satellites. From 10 to 18 h of incubation, the satellite spheroid moved towards and attached itself to the parental spheroid. After 24 h, a complete fusion between the satellite spheroid and the parental spheroid was observed. The same moving and attaching phenomena was observed in sharp micro-wells but at a faster rate as the complete fusion between the 1st and 2nd generation of spheroids took place after 8 h of incubation. Interestingly, the time-lapse images, as shown in Figure 5, indicate that even with confined circumstances, newly introduced cells did not clump together entirely on the existing spheroid but instead tended to form a satellite or secondary spheroid surrounding the parental spheroid. Furthermore, the newly formed secondary spheroid moved toward and eventually fused with the parental spheroid. The driving force underlying the interaction between the two generation spheroids requires further investigation.

### 2.3. Spheroid Migrating and Assembling between the Same Type of Tumor Cells

**Figure 6:**
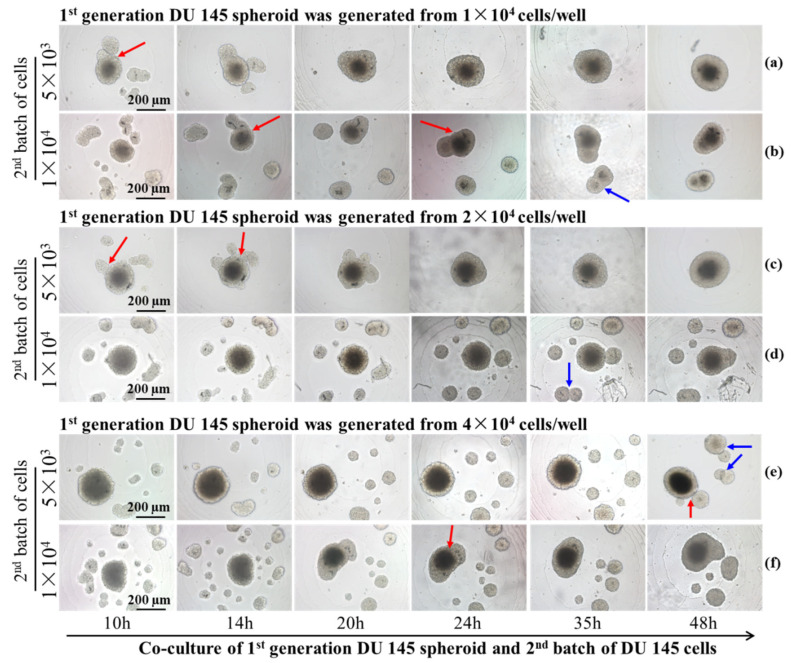
Time-lapse images of the interaction between two generations DU 145 tumor spheroids formed from different cell numbers. The parental spheroids formed from 1 × 10^4^ cells, and the 2nd batch cell number were 5 × 10^3^ (**a**) and1 × 10^4^ (**b**). The parental spheroids formed from 2 × 10^4^ cells, and the 2nd batch cell number were 5 × 10^3^ (**c**) and 1 × 10^4^ (**d**).The parental spheroids formed from 4 × 10^4^ cells, and the 2nd batch cell number were 5 × 10^3^ (**e**) and 1 × 10^4^ (**f**). The red arrows point to the fusion between parental and satellite spheroids, and the blue arrows point to the fusion between satellite spheroids. Scale bar = 200 µm.

The subsequent experiments utilized concave micro-well arrays to investigate the cell interactions between similar and dissimilar cell types. The primary objective was to systematically examine the interaction between the same and different types of cells. For interactions between 1st generation tumor spheroids and the 2nd batch of tumor cells, the influence of the parental spheroids’ initial cell number or volume, and consequently added cell number, on the overall dynamics of the two-generation tumor spheroids’ assembly and fusion were studied. In Figure 6a,b, the 1st generation DU 145 spheroids were formed from 1 × 10^4^ cells after 5 days of incubation. Then, 5 × 10^3^ and 1 × 10^4^ DU 145 cells were subsequently introduced, resulting in the formation of satellite DU 145 spheroids around the 1st generation spheroid. Contacting and fusion of the satellite and parental spheroid (red arrow) was observed after 10 h of co-incubation (Figure 6a). All newly added 2nd batches of DU 145 cells (5 × 10^3^ cells) fused with the parental spheroid after 20 h of incubation. By contrast, when the 2nd batch contained 1 × 10^4^ cells, satellite spheroids increased to seven by 10 h of incubation (Figure 6b). Some satellite spheroids drifted to the parental spheroid and fused with it (red arrow in Figure 6b). In addition, part of the satellite spheroids moved towards each other and eventually merged to form another spheroid (blue arrow in Figure 6b), and remained independent of the parental spheroid after 48 h of incubation.

The formation of satellite spheroids by the 2nd batch of DU 145 cells was observed from co-culture with parental spheroid generated from 2 × 10^4^ cells (Figure 6c,d). Complete fusion of the 1st generation spheroid and 2nd batch of 5 × 10^3^ cells was observed after 24 h of incubation (Figure 6c, Supplementary Video S1). However, the dynamics of movement and fusion for the second batch of 1 × 10^4^ cells were more distinctive. Most satellite spheroids moved toward their neighbors rather than drifted toward the parental spheroid. As a result, after 48 h of incubation, enlarged satellite spheroids maintained a certain distance from the parental spheroids (blue arrow in Figure 6d, Supplementary Video S2). The restrained or impeded merging and fusion with parental spheroids originating from 4 × 10^4^ cells are more apparent. Figure 6e, f indicate that most newly introduced 2nd batch of DU 145 cells (5 × 10^3^ cells and 1 × 10^4^ cells) formed satellite spheroids independent of the 1st generation parental spheroids (Figure 6e,f).

**Figure 7:**
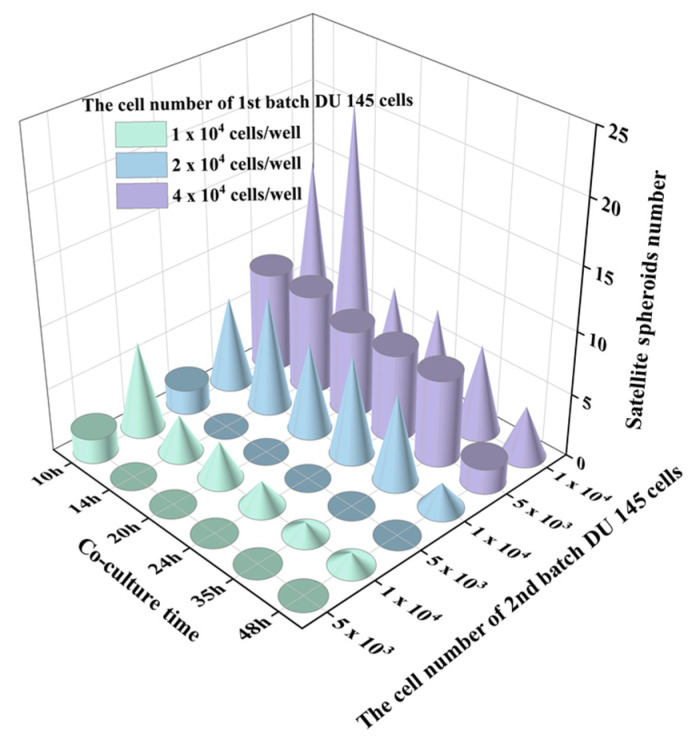
The number changes of satellite spheroids formed by a newly added 2nd batch of cells over time. The cylinders indicate the 2nd batch cell number as 5 × 10^3^ and the cones indicate the number of the 2nd batch cell number as 1 × 10^4^.

From the time-lapse images, we observed that the interaction of two-generation spheroids was affected by the cell number/density of both batches of cells. The number of satellite spheroids formed by the 2nd batch of cells was counted in Figure 7. It was found that the number of satellite spheroids decreased with the extension of the cultivation time. As the cultivation time increased, the satellite spheroids gradually migrated and fused to the 1st spheroid. Alternatively, when the volume of the parental spheroid and the number of the 2nd batch cells are large, the number of satellite spheroids formed by the 2nd batch of cells increased first and then decreased during the co-culture time. This phenomenon was most pronounced when the initial cell number of the parental spheroid was 4 × 10^4^ cells/well, and the number of the 2nd batch of cells was 1 × 10^4^ cells/well. The behavior of the 2nd batch of cells was associated with its cell number. The area supporting the cell growth and migration is constant. When relatively fewer cells were added (5 × 10^3^ cells), single cells followed Brownian motion and gradually gathered at the center of the micro-well. However, when high-density cells were added (1 × 10^4^ cells), the cell-cell distance was reduced, and the cell-membrane protein-mediated cell-cell aggregation dominated the cell behavior, resulting in satellite spheroid formation. The larger the volume of the parental spheroid, the smaller the area the 2nd batch of added cells could distribute. Therefore, the 2nd added cells are more likely to aggregate together to form satellite spheroids. When parental spheroids formed from a more significant cell number (2 × 10^4^ cells and 4 × 10^4^ cells), the cell-free space available for the 2nd cell batch was limited, resulting in prompt cell collision and the formation of satellite spheroids. In the following time, some satellite spheroids fused, and some attached to a parent spheroid, so the number of satellite spheroids gradually decreased.

### 2.4. Spheroid Migrating and Assembling with Different Cell Types

**Figure 8:**
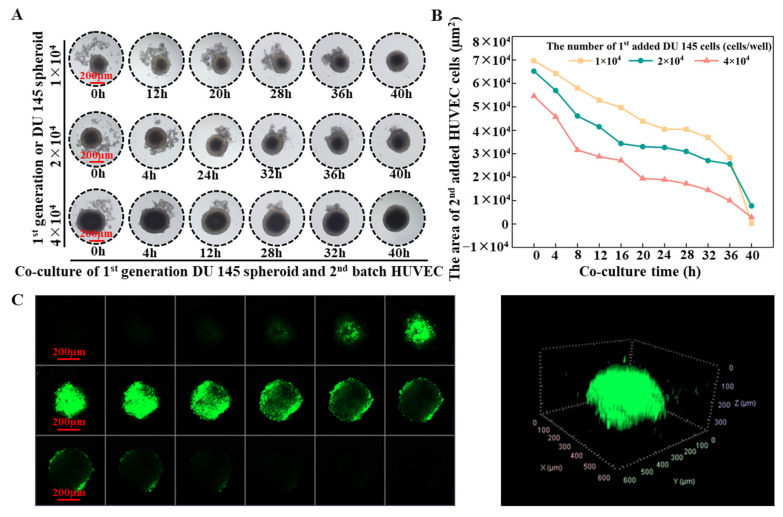
(**A**) Time-laps images of interaction between parental DU 145 tumor spheroids and subsequently added HUVECs. Scale bar = 200 µm. (**B**) Changes in the area of HUVECs over time. (**C**) Confocal images of the distribution of HUVECs in a tumor spheroid. Scale bar = 200 µm.

Tumor angiogenesis refers to the physiological and pathological process of blood vessel formation within solid tumors. To understand this process better, we studied the interaction between tumor spheroids and human umbilical vein endothelial cells (HUVEC) using concave agarose micro-wells. Figure 8A demonstrates that when 3 × 10^3^ HUVECs were added to the micro-well, containing tumor spheroids formed by varying cell numbers, HUVECs were observed to gather around the spheroids rather than aggregate on their own to form HUVEC spheroids. The HUVECs attached themselves to the existing tumor spheroids and penetrated the parental tumor spheroids (Supplementary Video S3). The fusion with the spheroids originating from 4 × 10^4^ tumor cells was completed after 28 h of incubation, while a slower fusion occurred with spheroids formed from 1 × 10^4^ cells. ImageJ analysis was applied to quantify the HUVEC distributed area. It was found that when HUVECs were added, part of them was attached to the parental tumor spheroid. The larger the volume of the parental spheroids, the more HUVECs that were attached to it (the smaller the HUVECs spreading area at 0 h), and the faster the HUVEC distribution area decreased over time. The rate of HUVEC area reduction, when encountering a tumor spheroid formed from 4 × 10^4^ cells, was significantly faster than that of a tumor spheroid formed from 1 × 10^4^ cells (Figure 8B). After the HUVECs had all entered the tumor spheroid, the distribution of the HUVECs within the tumor spheroid was characterized by confocal microscopy. A higher density of Dio-labeled HUVECs was observed from the inner core of the tumor spheroid (Figure 8C).

## 3. Discussion

Various tissue engineering strategies have been developed to meet the need to construct tissues or organisms with various functions in vitro. Among them, spheroid fusion is a relatively simple and convenient way. Spheroids undergo multiple levels of self-assembly in the process of fusion. The size of the agarose-based micro-well is 2 mm in diameter and 2 mm in depth, thus having a larger volume than the agarose micro-wells used in previous studies [33,37,38]. The larger size of the agarose micro-well makes it possible to precisely pipette the same amount of cell suspension into each well, ensuring cell number uniformity between the wells and the reproducibility of different batches. The previous studies focused on the fusion of spheroids that were direct contact with each other [14,19,26,28,33,39]. However, in this work, the larger micro-well was adopted to investigate the interaction between two generations of cells for the first time, observing the fusion of the contacted spheroids and the migration behavior of the 2nd generation cells before they contacted the 1st generation cells. By using the proposed concave bottom agarose micro-wells, we observed different fusion behaviors in two generations of cells and explored some macroscopic-level influencing factors. It was found that DU 145 cells are more inclined to form spheroids, while the HUVECs are not. The migration and fusion of tumor spheroids were determined by the size of the migration space and by the volume of the 1st batch of cells. Cells migrated and interacted after settling at the bottom of the well. The larger the space at the bottom, the more dispersive the cell distribution and the larger the distance between the cells. Therefore, it was more difficult for cells to communicate with each other, which resulted in irregular aggregate shapes and the slow migration of cells. Figure 3 and Figure 5 show that cells in the micro-wells with a flat bottom are irregular cell aggregates and have the slowest migration speed of the 2nd generation cells. The V-shape bottom micro-well has a comparatively limited space for cells to move. The spheroid formation of 1st generation cells and the migration of 2nd generation cells in V-shape bottom micro-wells were faster than those that took place in the concave bottom micro-wells. To depict the interaction between the two generations of cells, concave bottom micro-wells were applied in this study. Upon comparing the interaction between the spheroids and the cells depicted in Figure 6, it was observed that the volume of the parental spheroid and the number of newly added cells significantly determined the location and movement patterns of the resultant satellite spheroids when the same cell type was used. To observe the changes in the two generations of cells during the fusion process, especially the migration of the satellite spheroids formed from the second batch of cells, 1 × 10^4^ cells of the second batch of cells could be applied. Conversely, HUVECs, naturally attracted by cytokine secretion from tumor cells, exhibit a strong affinity towards attaching to a parental tumor spheroid. Figure 8 shows that the larger the primary cell spheroid volume, the faster the HUVECs migrate and penetrate into the spheroid. Previous studies have suggested that hypoxia-inducible factor (HIF)-1, which stimulates the up-regulation of vascular endothelial growth factor (VEGF), plays a crucial role in solid tumor expansion [40]. VEGF then attracts vascular endothelial cells to grow into the tumor and form microvasculature [41]. Larger spheroids may secrete more VEGF, and their more hypoxic core may synergistically attract HUVECs to penetrate the tumor spheroids. These observations indicate the importance of analyzing the biological properties of cells before designing experimental procedures for the sequential fusion of cell spheroids. It should be aware that the study was not performed on a biomimetic matrix, and the microenvironment provided by the cell culture medium in the agarose micro-wells was quite different from the microenvironment in vivo. Thus, the results obtained in this study should be further validated using animal and human-tissue based 3D tissue models [42]. Moreover, the stiffness of the substrate is essential for cell behavior, such as cell adhesion, differentiation, and migration [43,44,45]. A deep dive into the role of matrix stiffness for cell assembly needs to be taken in future studies. Moreover, the information presented in this study was limited to the available free space for subsequently added cells. Thus, more experiments are needed to understand how other factors, such as extracellular matrix, cell types, and cell-secreted factors, affect cell behavior and interactions.

## 4. Materials and Methods

### 4.1. Materials and Reagents

Agarose (for biochemistry, MW= 630.5, melting point 62–68 °C, PubChem CID 11966311, Catalog# A104062) was purchased from Aladdin, China. Human prostate cancer cells (DU 145) and human umbilical vein endothelial Cells (HUVECs) were bought from the Cell Bank of the Chinese Academy of Sciences (Shanghai, China). The cells were maintained in Dulbecco’s TM^®^Modified Eagle Medium (DMEM, Gibco, Gaithersburg, MD, USA) containing 10% fetal bovine serum (Gibco), penicillin (100 U/mL), and streptomycin (100 µg/mL) at 37 °C in a 5% CO_2_ atmosphere. Long-term fluorescence tracer Dio was purchased from Beyotime Biotechnology, Shanghai, China. Phosphate-buffered saline (PBS) was bought from Dingguo Biotechnology Company (Beijing, China). The distilled water was obtained from Millipore Q-Grad^®^1 system (Boston, MA, USA).

### 4.2. Design and Fabrication of Agarose Micro-Well Array

The mold replicating the agarose micro-well array was printed by a MoonRay UV DLP printer (SprintRay Inc., Los Angeles, CA, USA) with photosensitive resin (CLEAR SP-RH1004). As shown in Figure 1, the height of the U-shaped column in the mold is 2 mm, and the radius is 1 mm. The agarose solutions with different concentrations were prepared by mixing agarose powder and DI water. Then the mixture was placed in an autoclave. The agarose powder was completely dissolved in DI water during the autoclave process to form a homogeneous solution. An agarose solution of 700 µL was added to cover the 3D printed mold and allowed to solidify at room temperature (25 °C). Separating the agarose base from the mold carefully after the agarose had solidified, an agarose micro-well array with a depth of 2 mm and radius of 1 mm was obtained.

### 4.3. Characterization of the Mechanical Property of the Agarose Gels

Agarose gel cylinders (diameter = 27 mm, Height (H_0_) = 35 mm) of different concentrations were prepared for compression mechanics testing using Electromechanical Universal Testing Machine (CMT4503, MTS, Shanghai, China). The inlet force was set as 1 N, and the compression speed was 10 mm/min. The machine stopped when a relative drop in compression force was 80%. The resulting data was obtained, and then made a stress-strain curve. The stress and strain of the agarose gels were calculated as follows:

(1) $$\sigma =\frac{F}{A}$$

(2) $$\varepsilon =\frac{\Delta X}{H_{0}}$$

where F is the compression force (N) on the agarose cylinders, A is the surface area (m^2^) of the agarose cylinders, H_0_ (m) is the original height of the agarose cylinders, andΔX is the displacement of the compression plates. The values are expressed as the means ± standard error (n = 3).

### 4.4. Formation of Tumor Spheroids on Agarose Micro-Well Array

The DU 145 cells were harvested from a culture plate to prepare a cell suspension with different cell densities. The agarose micro-well arrays were placed inside a 12-well cell culture plate and 8 μL of the cell suspension was added to each agarose micro-well. Then a 600 µL cell culture medium was added to each well of the 12-well plate. The cells were cultured in a cell incubator (37 °C and 5% CO_2_), and the culture medium was changed every 3 days. The cell morphology was observed under a microscopic (TS100-F, Nikon, Japan). The number and size of the formed spheroids were characterized using the particle analysis function of ImageJ software V1.8.0 (NIH, Bethesda, MD, USA). To characterize the 3D structure of the spheroids formed on the agarose micro-well array, the spheroids were transferred to a tissue process cube and embedded in 1% agarose. After agarose gelling, the size of spheroids in the x-axis (top view) and the z-axis (side view) was observed under the microscope.

### 4.5. Migrating and Self-Assembly of Cells on the Agarose Micro-Well Array 

For migration and self-assembly between the cells of the same type, prostate cancer DU 145 cells were studied as a model cell. The first batch of DU 145 cells was seeded on an agarose array with different cell densities (1 × 10^4^ cells/well, 2 × 10^4^ cells/well, and 4 × 10^4^ cells/well). After 3 days of culture, spheroids were formed in each well. Under this premise, a second batch of cells with different numbers of DU 145 cells was added to wells that had been occupied by the first parental DU 145 spheroids of different volumes. The movement of the 2nd batch of cells was captured by the MonicCyte Living Cell Imaging System (MC-F100, Jiangsu Rayme Biotechnology Co., Ltd., Wuxi, China). In addition, the time lapse images of the interaction of the two batches of cells was recorded (TS100-F, Nikon, Tokyo, Japan). 

The DU 145 cells as a tumor cell model and HUVECs as an endothelial cell model were used for migration and self-assembly between cells of different types. To observe the clustering and distribution of the HUVECs, live cell fluorescent dyes Dio (10 μg/mL) was used to stain the cells according to the product protocols. In brief, the long-term fluorescence tracer solution Dio was added after the cells were collected in a 15 mL centrifugal tube. The cells were incubated at 37 °C for 10 min and then at 4 °C for 15 min. Next, the stained cells were washed 3 times with PBS (pH 7.4). Long-term fluorescence tracer Dio labeled HUVECs were adjusted to the required cell densities. During the co-culture experiment, the DU 145 cells (1 × 10^4^ cells/well, 2 × 10^4^ cells/well, and 4 × 10^4^ cells/well) were first seeded into the agarose micro-well. After 3 days of culture, parental DU 145 spheroids were formed. Then the second batch of cells (HUVECs with a cell density of 3 × 10^3^ cells/well) were added to the wells containing the DU 145 spheroids. The interaction of the DU 145 spheroids and the subsequently added HUVECs were observed and imaged under a microscope. Specifically, the HUVEC-occupied area was quantified by the image analysis function of ImageJ. When the supplemented HUVECs were completely fused with the parental DU 145 spheroids, the distribution of HUVECs in the tumor spheroids was characterized by a confocal laser scanning microscope (LSM 800, Zeiss, Germany). In detail, the samples were transferred to a 35 cm glass-bottom dish and scanned along the Z sequence with a step of 20 µm for a total of 18 slices. 

### 4.6. Statistical Analysis 

All experiments were performed three times. The data are expressed as the mean ± standard deviation. The experiment results were analyzed with the Student’s *t*-test using Origin Statistic software Version 9.8 (OriginLab, Northampton, MA, USA). *p* values of less than 0.05 were statistically significant.

## 5. Conclusions

In summary, the process of self-assembly in cells was studied using 3D tumor spheroids as a model. The behavior and movement of cells in response to variations in volume and density were studied. The findings indicated that the volume of parental spheroids and the number of newly added cells played significant roles in determining the location and movement of satellite spheroids. The results showed that when relatively fewer cells (5 × 10^3^ cells) were added, single cells followed Brownian motion, gradually moving towards the center of the micro-well and eventually fusing with the parental spheroid. A higher cell density (1 × 10^4^ cells and 2 × 10^4^ cells per well) promoted the formation of satellite spheroids. Sequentially adding tumor cells and HUVECs demonstrated the strong affinity of HUVECs (3 × 10^3^ cells per well) towards attaching to a parental tumor spheroid with different cell volumes (1 × 10^4^, 2 × 10^4^ and 4 × 10^4^ cells per well) and fusing with them. The results of this study highlighted the importance of understanding the biological properties of cells in designing experimental procedures for the sequential fusion of cell spheroids. The findings promoted a better understanding of the behavior and self-assembly of cells in 3D environments, which can enhance the development of more effective and personalized therapeutic strategies.

## Data Availability

The data in the current study are available from the corresponding authors upon reasonable request.

## Supplementary Materials

The supporting information can be downloaded at: https://www.mdpi.com/article/10.3390/ijms241511967/s1.
